# Importance of Venorrhaphy in Combined Popliteal Artery and Popliteal Vein Injury: A Case Report and Description of the Surgical Technique

**DOI:** 10.7759/cureus.87977

**Published:** 2025-07-15

**Authors:** Dasith K Jayawickrama, Joel Arudchelvam, Liyanage A Indunil, Deepthi I Kumari, Kugapiragash Rajkumar

**Affiliations:** 1 University Vascular and Transplant Surgery Department, National Hospital of Sri Lanka, Colombo, LKA; 2 University Vascular and Transplant Surgery Department, National Hospital of Sri Lanka, colombo, LKA

**Keywords:** arterial interposition graft, femoral supracondylar fracture, graft thrombosis, popliteal vein injury, venous hypertension

## Abstract

Popliteal vein (PV) injuries, which are less common than popliteal artery (PA) injuries, present with significant diagnostic and therapeutic challenges. These injuries are associated with a wide range of injuries. Early recognition and interventions provide better outcomes. Over time, the management of PV injuries has been shifted from ligation of the vessel to different forms of venous repair. We present a case of a 40-year-old female presenting with a supracondylar femur fracture with concomitant PA and PV injury. PA was repaired with a synthetic graft. However, the graft thrombosed due to venous hypertension. This was remedied with arterial graft thrombectomy and PV repair with an arterial interposition graft. This report highlights the importance of venous repair, especially in concomitant arterial and venous injuries.

## Introduction

Historically, popliteal vein (PV) injuries were commonly encountered on the battlefield. However, at present, PV injuries occur due to a range of causes, such as road traffic accidents (RTAs), violent encounters involving penetrating injuries and gunshot wounds, explosive fragment injuries, and blunt trauma [1]. Among the civilian and military populations, PV is the most common site of venous injury [2]. In particular, 37% of PV injuries have a concomitant popliteal artery (PA) injury [3].

Extremity venous injuries are often difficult to identify by physical signs only. Physical signs include hemorrhage, soft tissue swelling, pain, and features of compartment syndrome. Isolated venous injuries might not be symptomatic immediately after injury and may present later, i.e., with clinical features of deep vein thrombosis (DVT) [3].

If the patient is not in a life-threatening condition, imaging modalities including ultrasound scan (USS), computerized tomographic (CT) venography, and magnetic resonance imaging (MRI) could be used to facilitate the diagnosis [3,4].

Prior to the Korean war ligation of the PV was commonly practiced [5]. Thereafter, PV repair was increasingly practiced during the Vietnam war, due to the demonstrated benefits of the venous repair by several case series [6]. Subsequently, the venous repair was applied to venous injuries in the civilian population. Venous repair showed much improved limb salvage rates and less complications associated with venous ligation [7].

Long-term outcomes of lower limb vein repair are much better compared to ligation, especially in venous injuries of the popliteal vein and above [8-10]. In hemodynamically unstable patients and those with extensive soft tissue injuries, the optimal management of venous injuries remains a challenge, as the role of venous ligation continues to be necessary despite advancements in vascular repair techniques [11].

We present a case of a concurrent PA and PV injury that was noticed during an open reduction and fixation (ORIF). PA injury was repaired with a polytetrafluoroethylene (PTFE) graft, which failed due to the associated injury of the PV and venous hypertension. The PV was later repaired with a native interposition graft. The objective of presenting this case is to highlight the importance of venous repair and to describe the technique of using an arterial interposition graft in a venous repair.

## Case presentation

A 40-year-old female pedestrian presents following an RTA, with a left-side supracondylar femur closed fracture. The initial assessment demonstrated absent dorsalis pedis (DP) and posterior tibial (PT) pulses compared to the right side. No features of compartment syndrome were present. However, handheld Doppler ultrasound showed that there was a good flow in the DP and PT arteries. Further radiological imaging was not done, and it was decided to manage with close observation. For the femur fracture, an open reduction and internal fixation (ORIF) was planned.

During the ORIF, an abrupt pulsatile bleeding was encountered. A PA injury was suspected, and an immediate vascular exploration was done. 

**Figure 1:**
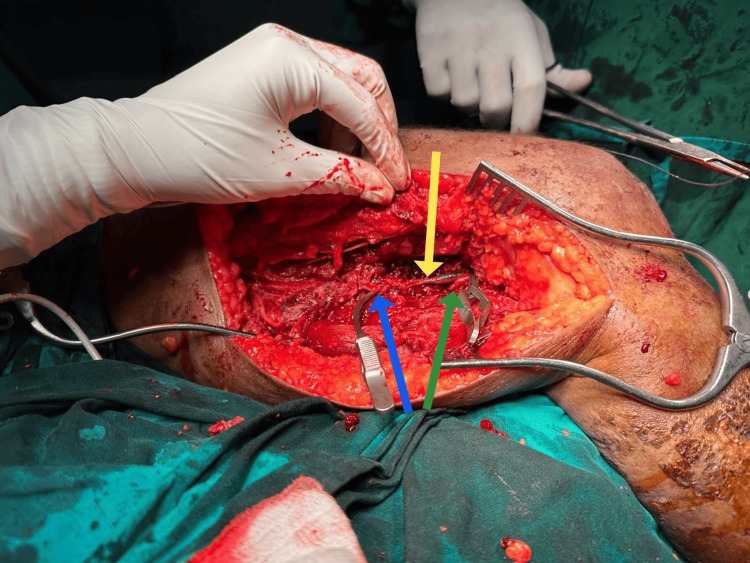
Injured popliteal artery and vein The proximal end of the popliteal artery is indicated by the blue arrow, and the distal end of the popliteal artery is shown by the green arrow. The yellow arrow shows the stenosed segment of the popliteal vein.

During the vascular exploration, the PA was found to have a laceration and a contusion about two inches inferiorly at the fracture site. The lacerated and the contused segments of the artery were trimmed (Figure 1). After trimming, the distance between the trimmed ends was widely separated. Therefore, an interposition graft repair was planned. The right greater saphenous vein (GSV) was found to be too narrow for a graft. Therefore, a 6 mm polytetrafluoroethylene (PTFE) graft was used, and anastomosis was constructed with a 6-0 polypropylene suture. Immediately following the repair, there was good distal pulsatile flow. Intravenous Heparin infusion was started postoperatively.

**Figure 2:**
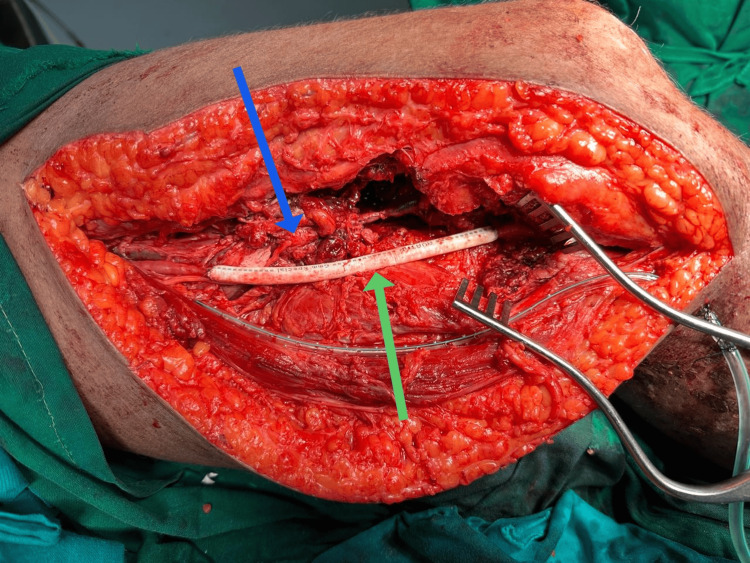
Following arterial repair The green arrow indicates the 6 mm polytetrafluoroethylene graft used for the interposition graft from healthier segments of the superficial femoral artery to the popliteal artery. The blue arrow indicates the segment of the artery proximal to the contused end, which was used as an arterial interposition graft to repair the popliteal vein.

**Figure 3:**
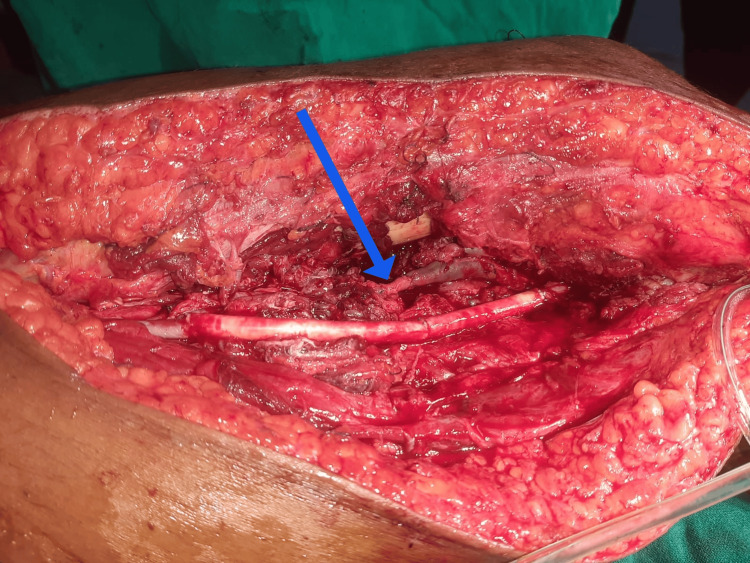
Following popliteal vein interposition graft repair The blue arrow indicates the arterial interposition graft used to repair the popliteal vein.

Four hours postoperatively, the distal (dorsalis pedis and the posterior tibial artery) pulses were not palpable. There were other features of acute limb ischemia as well. No flow through the graft was noted in the duplex ultrasound scan (USS). Therefore, a re-exploration was done. Arterial graft was found to be thrombosed. The PV near the knee joint was found to be distended. On further exploration of the PV, there was an injured narrowed segment on the PV. A thrombectomy of the PTFE graft on the artery was done with a 4F Fogarty catheter, and the distal limb arterial flow was restored. After restoring the distal arterial supply, the PV was found to be distending further, with high intravenous pressure (23 mmHg). Therefore, it was decided to repair the PV. The injured and narrowed segment of the PV was excised. After excision, the gap between the PV ends was more than 2 cm, and direct approximation was not possible without tension. The previously preserved segment of the PA between the contused and the lacerated segment was found to be adequate in length and diameter. Therefore, the arterial graft segment was used as an interposition graft to repair the defect of the PV with 6-0 polypropylene suture (Figures 2, 3). Postoperatively, intravenous heparin infusion was continued. The rest of the postoperative period was uneventful, with palpable distal pulses. The patient was kept on IV heparin infusion for two days and converted to aspirin afterward. The patient was discharged on postoperative day four.

**Figure 4:**
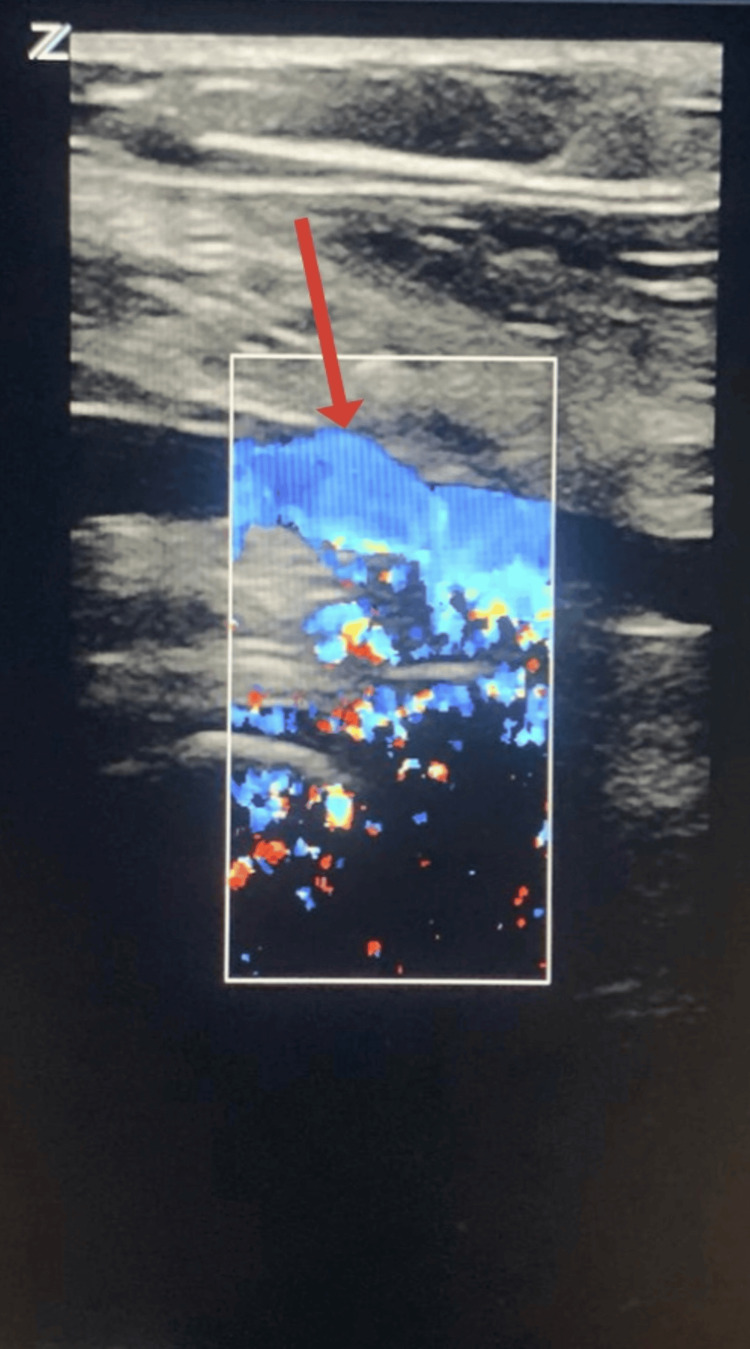
Ultrasound duplex scan of the venous repair site The red arrow shows the flow in the arterial interposition graft used to repair the popliteal vein.

At two weeks postoperative follow-up, there were no signs of surgical site infection, and USS showed good flow in the PTFE graft and the repaired PV (Figure 4). Aspirin was continued for two more weeks before being discontinued. The patient is currently being followed up at our clinic, and there are no functional or anatomical defects in the limb. 

## Discussion

Repair of PV injuries, unlike PA injuries, are not often documented in the literature. PV injuries make up most of the venous injuries, including civilian and military injuries [2]. PV injuries are associated with arterial injuries in 37% of cases [3]. Isolated venous injuries are often missed. The reason for this is that hematoma formation and edema are the most common early manifestations of venous injuries, but these signs are also common following trauma without venous injury [3].

Diagnosis is supplemented with imaging modalities such as USS, CT venogram, and MRI. USS, which is the most often performed initial imaging modality, is less sensitive and can only detect flow and thrombosis. By contrast, CT venography and MRI can demonstrate detailed anatomy of the venous tree and level of injury and associated soft tissue and bone injuries [3-5]. CT venography interpretation is difficult in explosive shrapnel injuries because of the artifacts caused by the shrapnel.

Until the early 20th century, the preferred method of treatment for PV injuries was ligation. For example, during the Korean war, 85% of the injured veins were ligated [5]. Since then, the value of venous repair has been demonstrated with improved limb salvage rates. At present, the venous repair is advised over ligation [6,7]. There is a wide range of methods of venous repair: lateral venorrhaphy, which is the first introduced method of repair by Hughes, end-to-end primary anastomosis, interposition graft repair with native (with spiral or panel reconstruction grafts) or prosthetic grafts, and venous patch angioplasty [7]. The long-term patency rates are lower in complex venous repairs, with almost 50% having early graft thrombosis [8]. It was demonstrated that injuries involving the popliteal vein and above respond well with venous repair compared to infra-popliteal veins [9]. Also, it has been demonstrated that the importance of venous repair in the background of concomitant arterial repair [10]. However, in the case of life-threatening situations, ligation of PV still has a place [11].

In our case, the PV stenosis and thrombosis resulted in venous hypertension and stasis causing the synthetic graft arterial repair to thrombose. We opted for an interposition graft for the PV repair because the resected vein length was about 2 cm and an end-to-end repair was not possible without tension. We opted for arterial interposition graft because we noticed a healthy segment of the artery that was no longer part of the main arterial flow to the limb and the native veins were not suitable for an interposition graft.

## Conclusions

PV injuries are increasingly encountered in the civilian and military populations due to the increasing trend of violent injuries, global conflicts, and terrorism. Early diagnosis and management of venous injuries can reduce patient morbidity and improve limb salvage rates. The use of imaging modalities will facilitate the diagnosis and should be used in cases of doubt. With the current evidence, it is clear that venous repair has much better outcomes compared to plain ligation. As in our case, in the background of a PA repair, a concomitant PV injury should be repaired for better outcomes.
